# The fifth cranial nerve in headaches

**DOI:** 10.1186/s10194-020-01134-1

**Published:** 2020-06-05

**Authors:** J. C. A. Edvinsson, A. Viganò, A. Alekseeva, E. Alieva, R. Arruda, C. De Luca, N. D’Ettore, I. Frattale, M. Kurnukhina, N. Macerola, E. Malenkova, M. Maiorova, A. Novikova, P. Řehulka, V. Rapaccini, O. Roshchina, G. Vanderschueren, L. Zvaune, A. P. Andreou, K. A. Haanes, On behalf of the European Headache Federation School of Advanced Studies (EHF-SAS)

**Affiliations:** 1 Department of Clinical Experimental Research, Glostrup Research Institute, Rigshospitalet Glostrup, 2600 Glostrup, Denmark; 2 Department of Drug Design and Pharmacology, Faculty of Health and Medical Sciences, University of Copenhagen, Copenhagen, Denmark; 3 IRCCS Fondazione Don Carlo Gnocchi, Milan, Italy; 4 Department of Neurology, First Pavlov State Medical University of St.Petersburg, St.Petersburg, Russia; 5 GBUZ Regional Clinical Hospital № 2, Krasnodar, Russia; 6 Department of Neuroscience, University of Sao Paulo, Ribeirao Preto, Brazil; 7 Department of Clinical and Experimental Medicine, Neurology Unit, University of Pisa, 56126 Pisa, Italy; 8 Department of Public Medicine, Laboratory of Morphology of Neuronal Network, University of Campania-Luigi Vanvitelli, Naples, Italy; 9 Department of Neurology, University of Rome, Tor Vergata, Rome, Italy; 10 Department of Applied Clinical Sciences and Biotechnology, University of L’Aquila, 67100 L’Aquila, Italy; 11 Department of Neurosurgery, First Pavlov State Medical University of St.Petersburg, Lev Tolstoy Street 6-8, St.Petersburg, Russia; 12 The Leningrad Regional State Budgetary Institution of health care “Children’s clinical hospital”, St.Petersburg, Russia; 13 Department of Internal Medicine, Fondazione Policlinico Universitario Agostino Gemelli IRCCS Università Cattolica del Sacro Cuore, Rome, Italy; 14 Pain Department, Petrovsky National Research Centre of Surgery, Moscow, Russia; 15 Faculty of Medicine, University of Tartu, Tartu, Estonia; 16 F.F. Erisman Federal Research Center for Hygiene, Mytishchy, Russia; 17 Department of Neurology, St. Anne’s University Hospital and Faculty of Medicine, Masaryk University, Brno, Czech Republic; 18 Child Neurology and Psychiatry Unit, Systems Medicine Department, University Hospital Tor Vergata, Viale Oxford 81, 00133 Rome, Italy; 19 Unità Sanitaria Locale (USL) Umbria 2, Viale VIII Marzo, 05100 Terni, Italy; 20 Department of Neurology, Headache Center, Ospedale Pediatrico Bambino Gesù, IRCCS, Rome, Italy; 21 Department of Neurology, ZNA Middelheim, Lindendreef 1, 2020 Antwerp, Belgium; 22 Department of Anaesthesiology and Intensive Care, Faculty of Medicine, Riga Stradins University, Riga, Latvia; 23 Department of Pain Medicine, Hospital Jurmala, Jurmala, Latvia; 24 Headache Centre Vivendi, Riga, Latvia; 25 Headache Research, Wolfson CARD, Institute of Psychiatry, Psychology and Neuroscience, King’s College London, London, UK; 26 The Headache Centre, Guy’s and St Thomas, NHS Foundation Trust, London, UK

**Keywords:** Fifth cranial nerve, Trigeminal ganglion, Headache, CGRP, Treatments, Migraine pathophysiology

## Abstract

The fifth cranial nerve is the common denominator for many headaches and facial pain pathologies currently known. Projecting from the trigeminal ganglion, in a bipolar manner, it connects to the brainstem and supplies various parts of the head and face with sensory innervation. In this review, we describe the neuroanatomical structures and pathways implicated in the sensation of the trigeminal system. Furthermore, we present the current understanding of several primary headaches, painful neuropathies and their pharmacological treatments. We hope that this overview can elucidate the complex field of headache pathologies, and their link to the trigeminal nerve, to a broader field of young scientists.

## Introduction

Considering that the classification of headache disorders (ICHD-3) contains almost 300 different types of headaches and facial pains [1], it is quite surprising that a large part of pathophysiological mechanisms rely on the same anatomical basis. Since the early work by *Harold Wolff* and his contemporaries [2] it has been shown that, among intracranial structures, only the dura mater, its vessels and the cerebral blood vessels are pain sensitive and can show referred pain on various extracranial positions [3]. This classical view was recently expanded by an observational study [4] to include pia mater and its cortical arterioles as potential pain sensitive structures. Subsequent neuroanatomical and neurochemical studies revealed that most sensory fibres from the intracranial and the extracranial tissues originate in the fifth cranial nerve (CN V) ganglion, also called trigeminal ganglion (TG). However, not all intracranial sensory fibers are trigeminal. For example, the posterior cranial fossa, is mainly innervated by the occipital nerves.

Depending on which part of the head is innervated the fibres can be traced back to different parts of the TG [5]. In general, headache pain is referred to a cutaneous territory area on the scalp, sharing supply with a nerve innervating the intracranial area, which might be the actual source of pain. Similarly, pain can be referred to a different territory than the actual nerve receiving the painful stimulation. This can happen if the two nerves share a high-order neuron (a process called “convergence”).

**Fig. 1:**
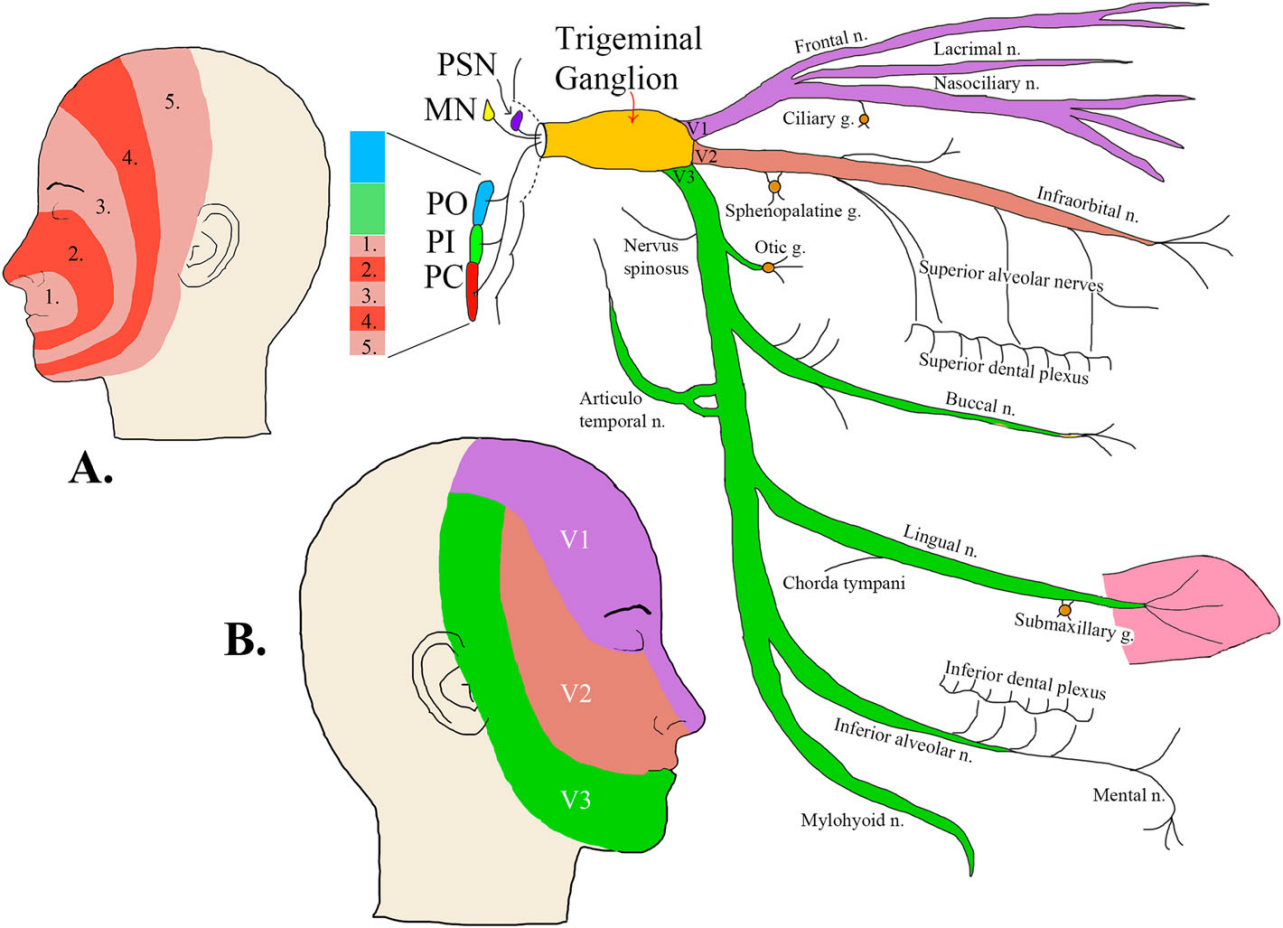
Schematic of the Trigeminal System. **a**: The somatotopic distribution of trigeminal nociceptive afferents terminating in the trigeminal nucleus caudalis [11]. **b**: Innervation of facial skin areas and its related three branches (V1, V2 and V3). PSN (Principal sensory nucleus CN V), MN (Mesencephalic nucleus CN V), PA (Spinal nucleus of CN V Pars Oralis), PI (Spinal nucleus of CN V Pars Interpolaris), PC (Spinal nucleus of CN V Pars Caudalis). N. = Nerve. G. = Ganglion

Primary headaches comprise the most prevalent group of neurological disorders. Among these, migraine is estimated to be present in 14.4% of the global population [6]. The WHO ranks migraine as the most prevalent, disabling, long-term neurological condition when taking into account years lost due to disability in young individuals [7, 8]. The burden on individuals and society is enormous [9], especially if other headaches such as tension-type (TTH), the second more common disorder worldwide [7, 8], and medication-overuse headache (MOH) are taken into account. Though TTH is more prevalent (26.1%) [6], migraine is the more debilitating, as migraine has been reported to contribute 16.3% of disability-adjusted life-years on the global burden of neurological disorders [10]. The present work is a comprehensive description of various aspects of the CN V, the largest of the cranial nerves. Its more common name “trigeminal” (triplet) derives from its clearly visible division into three main branches (Fig. 1). In this review we explore the trigeminal nerve, its related pain conditions and current treatments to emphasize its importance to headache pathophysiology.

## The Trigeminovascular system

The vascular system of the head, face, meninges and the brain have a variable innervation of autonomic and sensory nerves [12]. In general, the arterial system is richly supplied with sensory nerves whereas the veins are weakly innervated. Capillaries are not innervated. For the cerebral vasculature, it is different; while the pial or extracerebral arterial system is richly supplied, once the vessels penetrate into the brain parenchyma their autonomic and sensory fibres disappear (at the level of the space of Virchow), as these are regulated by metabolic demand [13].

The trigeminovascular system has long been a focus of elucidating primary headache pathophysiology [14]. It consists of the trigeminal neurons innervating the cerebral arteries, the pial and dural blood vessels, and sinuses [15]. Nociceptive activation of C- and Aδ-fibres innervating these structures is thought to be involved in the headache phase of migraine. The cranial dura mater nerve fibres are mainly supplied by the ophthalmic branch (V1), though collaterals from the maxillary branch (V2), the mandibular branch (V3) and cervical root ganglion provide dural innervation to smaller caudal regions. Afferents from the TG carry this nociceptive information into the brainstem where they mainly terminate at second order neurons inhabiting the trigeminocervical complex (TCC) [15].

Studies have shown that parts of the trigeminovascular system (notably TG) lack blood-brain barrier (BBB) and has been hypothesized being the target tissue where some anti-migraine drugs (e.g. monoclonal antibodies, gepants, triptans) elicit their effects [16]. Because of this evidence it is likely that CN V is an integral part in understanding headache pathophysiology [17].

### The trigeminal ganglion

**Fig. 2:**
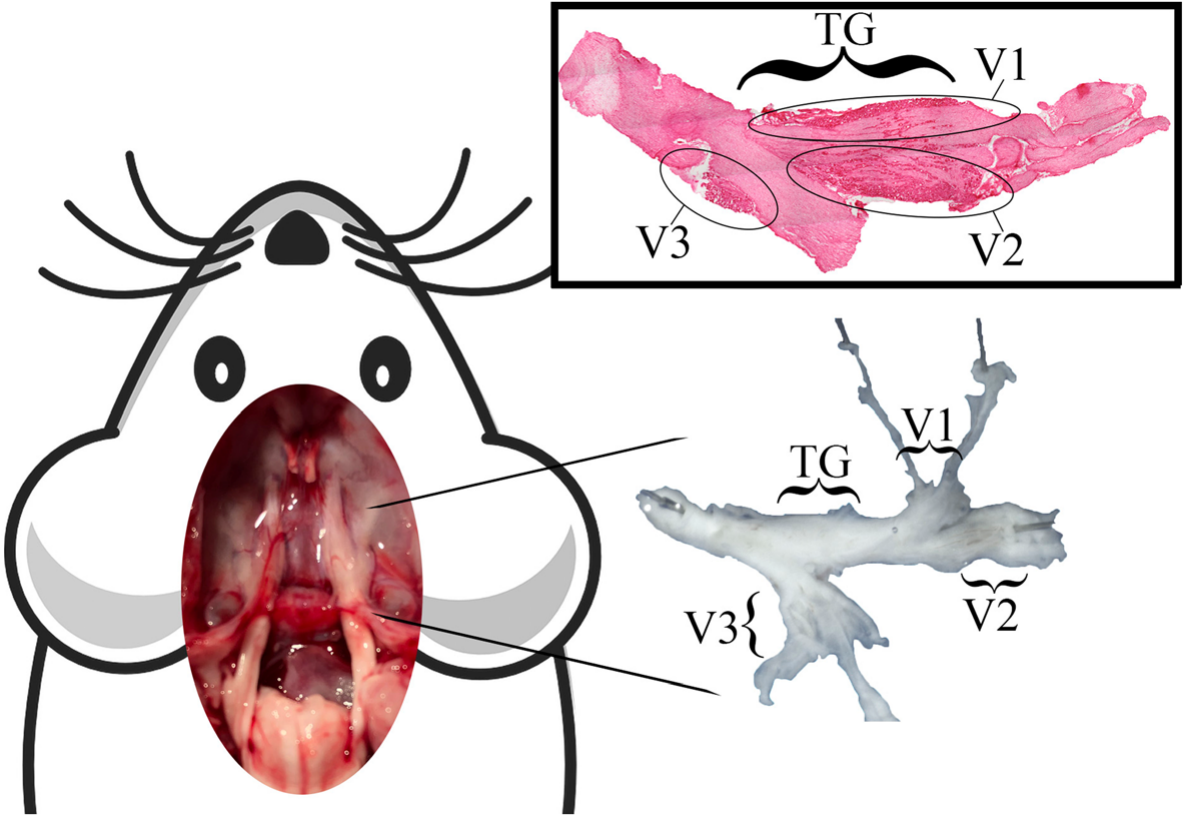
**Left**: Superior perspective of a dissected male rat displaying both Meckel’s caves containing the trigeminal ganglion (TG). **Right**: TG and parts of its main branches. **Insert**: Hematoxylin-eosin staining of rat TG displaying neuron (darker areas) locations and their approximate branch affiliation

Shortly after CN V protrudes from each side of the superior lateral pons the TG can be found residing in each of Meckel’s caves (Fig. 2). The TG has been termed a “central hub” in the trigeminovascular transmitting pathway as it contains the soma of the peripheral nerves able to activate superior order sensory neurons inhabiting the TCC which in turn progress the signal to the thalamus and finally cortex.

Interestingly, in an experimental rat study [18], injection of a retrograde tracer in various facial regions could be traced to specific neuron clusters in the TG. The injections were made into regions correlating to the V1 (eyebrow or directly on the eye), V2 (whisker pad) and V3 (temporomandibular joint capsule). When examined, the tracer could be located in TG neuron somas revealing an organisation of the ganglion and a potential origin for each branch (Fig. 2) [18, 19].

The pseudo-unipolar neurons of the TG are implicated in a variety of sensory and nociceptive stimuli, in the craniofacial region, including mechanical, chemical and thermal inputs [20, 21]. In the TG, small neurons process the action potentials of peripheral noxious stimuli carried by afferent C- (unmyelinated) and Aδ-fibres (thinly myelinated), which are believed to convey the nociception of headache [14, 21]. The larger diameter, myelinated fibres are, on the other hand, mainly responsible for tactile stimulation processing [22] and also known as low-threshold mechanosensitive afferent Aβ-fibres [23–25] that normally do not mediate pain [23].

Applying microscopy, many morphological features of the TG have been described including: the composition of the neuron-glia unit (NGU), nerve bundles and extracellular matrix with microvessels, nerve fibres, together with occasional mast cells and stromal cells [26] that are all dependent on each other [27]. The NGU consists of one to three neuronal cells enveloped by a discontinuous sheath of satellite glial cells (SGC) [26]. Electron microscopy further shows that Schwann cells comprising the myelin sheath combined with microvessels with endothelial cells and to some extent pericytes. The absence of astrocytes could explain why the TG lacks a proper BBB [14, 21, 26].

The sensory fibres innervating intracranial vessels have their origin in the TG and they store a number of neurotransmitters, with the most prominent being glutamate, and neuropeptides, including dynorphins, Calcitonin Gene-Related Peptide (CGRP), serotonin, amylin, substance P, neurokinin A/B, Pituitary Adenylate Cyclase-Activating Polypeptide (PACAP). Receptors for these signalling molecules are expressed on peripheral and central structures, and importantly on the TG neurons themselves [14] (for a more detailed review see [28]). Furthermore, these signalling molecules are pivotal in cellular communication for pain processes (e.g. induction or central/peripheral sensitization), and therefore also in headache perception.

Migraine and cluster headache pain seem to rely on the CGRP pathway in the trigeminovascular system as vastly demonstrated (for a review on the subject see [14, 29]). As the TG is lacking a proper BBB [30], and has a high density of CGRP receptors accessible for anti-migraine drugs (e.g triptans and anti-CGRP directed drugs), the TG could represent a common denominator for headache and craniofacial pain processing and a preferential target for treatments.

The SGCs form a cellular layer covering almost all sensory neurons in the TG and similar to the astrocytes of other regions of the nervous system, ensure metabolic support, glutamate regulation and ionic balancing. The role of SGCs in neuropathic pain has been shown to be involved in the sensorial malfunctioning leading to “maladaptive” plasticity [31–35] that are responsible for chronification process widely described in common forms of headache (e.g. migraine) [36].

In particular, the regulation of extracellular potassium concentrations and consequently the utilization of ATP by specific ATP-ase pumps seems to be one of the regulator mechanisms of pain attributed to SGCs [32, 37]. Also, SGCs could modulate the purinergic system in the TG through vesicular nucleotide transporters (VNUT) [38]. In fact, all the purinergic receptors are expressed in the TG [39]. Purinergic signalling has two-sided effects in the TG. ATP release and the following purinergic activation, after peripheral noxious perineural stimulation, activate both SGCs and neurons in TG [34, 38]. This contrasts to the breakdown product ADP, which instead leads to trigeminovascular deactivation [40].

## Anatomy of the fifth cranial nerve

### The ophthalmic branch (V1)

The V1 nerve is the first branch of the trigeminal nerve in rostral-caudal order. From the TG it crosses the sidewall of the cavernous sinus and then passes through the superior orbital fissure into the orbit, where it divides into three terminal branches: the lacrimal nerve, the frontal nerve and the nasociliary nerve. These large branches in turn branch off to form smaller and ultimately terminating sensory nerves (e.g the frontal nerve branches off to form the supratrochlear nerve while the nasociliary nerve branches off to form the infratrochlear nerve and the anterior ethmoidal nerve, the latter further forming external nasal nerves).

The V1 is a sensory nerve that innervates the upper part of the face and the two thirds of the anterior scalp, from the level of the palpebral fissures to the area of ​​the coronal suture [41]. The terminals of the lacrimal and nasociliary branches provide the somatic sensation from the eye structures, so that damage of these nerves impair the corneal reflex. The V1 branch provides both superficial and autonomic sensory innervation to the ciliary body, lacrimal gland, conjunctiva, cornea and iris, albeit they do not originate from the trigeminal nucleus but from the superior cervical ganglion and the sphenopalatine ganglion (SPG). The former provides sympathetic fibres for the dilatator pupillae that run in the nasociliary branch, the latter provides parasympathetic fibres for the lacrimation partially running in the lacrimal branch of V1 [42, 43].

Furthermore, V1 supplies intracranial structures sensitive to pain, the superior part of the nasal cavity, medial orbital roof, crista galli, and the dura mater meninges, cerebral arteries in the circle of Willis [44], and, through the tentorial nerve of Arnold, reaching the traverse and straight venous sinuses [45]. Noxious stimuli to the intracranial sensory receptors are transduced predominantly by the ophthalmic branch because the maxillary and mandibular branches, or cervical dorsal root ganglia, provide the innervation of only a limited extent of the meninges [46–50]. This likely explains why the majority of headache present as painful sensation in this territory.

### The maxillary branch (V2)

The V2 nerve is the second branch of the trigeminal nerve. It reaches intracranially the dura of the middle cranial fossa, the upper teeth and the related oral gingiva, the palate and mucous membranes of the maxillary sinuses and nasal cavity [51]. Postganglionic parasympathetic neurons from the SPG (innervated by TG fibres) reach the lacrimal gland trough V2 branches, where they mix with homologous fibres coming from V1. Similarly, sphenopalatine branches supply intramural glands of the nose and the hard palate.

As a sensory nerve, V2 innervates the skin of the lower eyelid, the sides of the nose, nasolabial fold, upper lip and the cheek.

### The mandibular branch (V3)

The V3 nerve is the largest of the three branches of the trigeminal nerve in humans. V3 passes between *tensor veli palatini* and lateral pterygoid and gives off a meningeal branch (nervus spinosus, so called because it passes through the foramen spinosum) and the nerve to medial pterygoid from its medial side. The continuation of the mandibular nerve then splits into an anterior and a posterior trunk. The anterior trunk gives off branches to three major muscles of mastication and a buccal branch, which provides sensory innervation to the cheek. The posterior division gives off three main sensory branches, the auriculotemporal, lingual and inferior alveolar nerves and motor fibres to supply mylohyoid and the anterior belly of the digastric muscle [52].

The V3 branch innervates a territory of skin covering the posterior part of the temporal region, the anterior part of the earlobe, the anterior and superior walls of the external ear canal, the lower lip and the chin. Its mucosal territory covers the anterior two-thirds of the tongue, the medial aspect of the cheek and the floor of the oral cavity, the gingiva, and the mandibular alveoli and teeth. As previously mentioned, the V3 branch also carries trigeminal motor fibres that innervate the masticatory muscles (masseter, temporal, internal and external pterygoid, mylohyoid, anterior body of the digastric and the tensor palati) controlling biting and chewing mechanisms [53].

### The Trigeminocervical complex (TCC)

The first order sensory neurons of the TG project centrally to the trigeminocervical complex (TCC) in the brainstem. The TCC includes the second order neurons of the trigeminal sensory pathway inhabiting the trigeminal nucleus caudalis (TNC) and C1 and C2 segments of the cervical spinal region [54]. While historically considered as two separate entities, recently the trigeminal system has been considered both as a morphological [5] and a functional ensemble with first cervical roots [55]. The part of the TNC dedicated to pain perception is the lower part, the Pars Caudalis (PC), while rostral parts are mainly deputed to tactile perception. This pain specific part of the TNC extends from C2 or C3 rostrally to the level of the obex. The TNC has many cytoarchitectural similarities with the posterior horn. For this reason, it has been termed “medullary posterior horn” and has been divided into layers that correspond to Rexed spinal cord laminae [56]. The TNC and the posterior horn also show homology in the distribution of neurotransmitters; substance P and CGRP are localized in nociceptive C-fibres that terminate in both of these areas [56]. The most superior area of the TNC is the inferior medulla and the most inferior area is the upper cervical spinal cord [57]. The spinal trigeminal nucleus is a sensory tract located in the lateral medulla of the brain stem and descends to the caudal end of the medulla and into the spinal cord (as far as the third or fourth cervical level), where it becomes continuous with Lissauer’s tract [58] and takes sensory information from different cranial nerves, including the trigeminal nerve and its branches [54].

The innervation of the face forms a somatotopic map in the TNC, which is stretched and distorted into the proportions of the PC of the spinal trigeminal nucleus (Fig. 1a). The area of lips and perioral area constitute the outermost layer of the onion meaning that they lie within the most superior area of the TNC [57]. The next innermost layer lies inferiorly within PC and comprises the projections of nose, eyes, and outer oral areas. The V1 branch travels in the most ventral part of the spinal tract and extends caudally. The V2 branch lies in the most dorsal part of the trigeminal nucleus caudalis and terminates in the most rostral level [54]. In the lowest part, there are areas reserved to cheeks and forehead; then the vertical area of the ears; and finally the partial sensory innervation of the external ears (from cranial nerves VII, IX, and X) [57]. This pattern of termination may account for the onion skin pattern of facial sensory loss with intramedullary lesions [54]. The TNC that runs medial to the spinal trigeminal tract, also has an onion skin somatotopy, and divides into three different cytoarchitectural regions: Pars Oralis (PO), Pars Interpolaris (PI) and PC. PO is the most superior nucleus, running from the pons to the mid-medulla. PI is the middle nucleus, spanning in the mid-medulla. PC is the most inferior nucleus from the lower medulla to the upper cervical spinal cord. Its inferior extent is variably listed from C2 to C4 [57].

Pradier and McCormick reported in their study, based on electrophysiological characteristics of neurons of the TNC, that there are five main groups of neurons, including; tonic, phasic, delayed, H-current and tonic-phasic neurons, groups that exhibit distinct intrinsic properties and share some similarity with groups identified in the spinal dorsal horn [59]. The primary function of the TNC is to carry information on temperature, deep or crude touch (PO and PI), and pain from the portion of the face (PC) [54]. Afferents from the TNC terminate at third-order neurons inhabiting the thalamus (mainly posterior and ventral posteromedial thalamic nuclei) [58, 60].

In addition to this major pathway, TCC is also responsible for conveying sensory and nociceptive signalling from the meninges and craniovascular structures to several higher order relays. There are numerous direct ascending connections within the medulla (e.g. medullary pontine nuclei including the rostral ventromedial medulla), brainstem (e.g. nucleus raphe magnus, parabrachial nucleus and locus coeruleus), midbrain nuclei (e.g. ventrolateral periaqueductal gray and cuneiform nucleus), and diencephalon (e.g. hypothalamus and thalamus) [54, 58].

Activation of these structures are believed to contribute to the perception of pain during migraine, and also to autonomic, endocrine, cognitive and affective symptoms that last throughout the migraine episode [54]. Furthermore, the second order neurons receive inputs from the occipital nerve. This convergence may have treatment implications for some primary headache conditions as well as referred pain.

### Trigeminohypothalamic tract and the parabrachial-limbic tract

Although a detailed description of these relay-functions lies outside the purpose of the present review (for details see [61, 62]), we will briefly discuss the trigeminohypothalamic tract and the parabrachial-limbic tract.

The trigeminohypothalamic tract originates from specific nociceptive, multimodal intensity-coding wide dynamic range (fundamental for pain “gating effects”) and non-nociceptive neurons, albeit about the 80% of its fibres are axons from nociceptive neurons [61]. The trigeminohypothalamic tract ascends contralaterally in the brainstem but about half of the fibres present a decussation in the lateral hypothalamus, reaching both lateral and medial structures of hypothalamus (e.g. prefornical, suprachiamatic, supraoptic nuclei). While non-nociceptive information are transmitted only by direct pathway, nociception is carried both directly and indirectly (i.e. trigeminoreticular tract) to hypothalamus, suggesting a more resistant mechanism to pathological noxae for nociception [62]. Receiver areas of the hypothalamus are those regulating homeostasis and integrating pain with visceral afferent input [63].

The trigeminoparabrachial tract is a polysynaptic pathway connecting CN V to the limbic system, with direct tracts ending in the amygdala, lenticular nucleus, nucleus accumbens and it is thought to exert, among other functions, the transmission of visceral pain and the emotional value of pain sensations [62, 64]. The parabrachial nucleus, in fact, contains a large share of neurons expressing both CGRP and PACAP, especially in its lateral portion, which is the one activated by painful stimulation [65–67]. The transmission of CGRP is thought to reach directly the limbic system, where it can mediate aversive behaviour or freezing, as demonstrated in mice with injection of CGRP into the insula region [68].

## The trigeminal system in primary headache conditions

In the headaches most commonly seen in specialized units (e.g. migraine, TTH, trigeminal autonomic cephalalgias (TACs)) the pain generating mechanism resides in the complex relationship between trigeminal system and intracranial structures sensible to pain (mostly vessels and meninges). For this reason, the functional ensemble of the trigeminovascular system is quite relevant for the understanding of head and facial pain pathophysiology. We will now continue to describe some of the more notorious headache and facial pain diagnoses.

Other less common primary headaches, which are not explored in this article, that also might affect the trigeminal area are: primary stabbing headache (*head pain* occurs as a single *stab* or as a series of stabs*)*, nummular headache (characterized by small circumscribed areas of continuous pain on the head), cold-stimulus headache (a direct result of the rapid cooling and rewarming of the capillaries in the sinuses leading), and external pressure headache (external pressure on pain receptors or pain fibres) [1].

### Tension type headache

As previously mentioned, TTH is the most prevalent primary headache [23, 69]. The definition of TTH can be defined as a mild to moderate bilateral headache with a steady non-pulsating pain which is unaffected by movement and lasting 30 min to 7 days [70]. Furthermore, TTH is not associated with nausea or vomiting and can manifest with either photophobia or phonophobia. The pathophysiological mechanism of TTH is not yet completely clear and most likely multifactorially determined. Central sensitization of the trigeminal nerve seems to play an important role, especially in patients with chronic TTH.

Patients with episodic and chronic TTH have a considerably increased tenderness to palpation of pericranial myofascial tissues [71]. This increased tenderness originates from muscles, fascia and tendons throughout the pericranial region, probably due to sensitization of Aδ- and C-fibres [71]. After a strong peripheral nociceptive stimulus from pericranial myofascial tissues central sensitization can occur; ineffective synapses can change to effective contacts of low threshold mechanosensitive afferent nerves and superficial second order nociceptive neurons in the trigeminal nucleus, which usually receive input from high threshold mechanoreceptors [25]. This central sensitization may make innoxious stimuli more aggravating to the pain modulating systems, resulting in allodynia and hyperalgesia [23, 71].

### Migraine

In migraine, intracranial vasculature is innervated by trigeminal fibres (see above for details). Intracranial sensory receptors cover the rich plexus of meningeal perivascular nerves of pial and dural blood vessels. Currently, this classical vascular theory, naming vasodilation as the migraine pain generator, seems less reliable compared to a theory focusing on central sensitization, where activation of neuronal receptors is pivotal as origin of migraine pain. The current paradigm is supported by evidence of hypothalamic activation [72] and the view of vasodilation as an epiphenomenon rather than a causation of pain [73–75]. Nevertheless, the current review does not focus on the origin of the migraine attack, but the origin of the perceived pain, which most likely resides in the TG and the associated sensory fibers.

Whichever the trigger, the repeated experimental activation of the TG leads to release of vasoactive neuropeptides, such as substance P, CGRP [76] and PACAP [77]. CGRP and several other substances have been shown to evoke headaches after intravenous administration both in healthy subjects and migraineurs, and to trigger a delayed migraine-like attack in the latter group [78]. The release of vasoactive neuropeptides from the peripheral terminals of trigeminal nerve may result in neurogenic vasodilatation, plasma extravasation and trigeminal nerve sensitization, at least in rodents. It remains questionable whether the throbbing quality of migraine pain and aggravation by head movements or routine physical activity are expression of peripheral [79] or central sensitization process [46].

In addition to the importance of the neuropeptide signalling, some of the transient receptor potential (TRP) channels, which are identified in trigeminal ganglion, vagal ganglia and in dorsal root ganglia could play an important role. Those superfamily of receptors expressed in the trigeminal ganglion are mainly TRP vanilloid 1 (TRPV1) [80] and TRP ankyrin 1 (TRPA1) channels which might be involved not only in pain initiations but also as future treatment-targets in migraine [81]. TRPM8, which mediates the cold sensation has also been linked to migraine pathophysiology by genome wide association studies (GWAS), especially in the northern population through a mechanism of evolutionary selection [82, 83].

### Chronification, sensitization and habituation

Migraine is a progressive disorder and can transform from an episodic state to a chronic state. According to ICHD-3 [1], chronic migraine (CM) is defined as the presence of headache for 15 or more days/month for at least 3 months with migraine associated symptoms. A well-accepted mechanism driving the progression from episodic to CM is the peripheral sensitization of the primary afferent TG neurons, which leads to central sensitization of TNC second-order neurons and ultimately to central sensitization of third order neurons in the thalamus [46, 84].

In a first phase of activity-dependent central sensitization, the TNC neurons, under repetitive, persistent nociceptive stimuli from the TG, become sensitized and produce exaggerated and prolonged responses to lower threshold stimuli. Over time, a neuroplastic adaptation in medullary and cortical pain areas causes a shift in the pain modulatory system creating a new threshold and favouring a net pain facilitation rather than pain alleviation. This shift to activity-independent central sensitization plays a crucial role in the conversion to CM [85, 86].

Based on experiments by Burstein et al, it is hypothesized that cutaneous allodynia serves as a clinical indicator of migraine chronification. Particularly, the development of cutaneous allodynia in the head indicates that central sensitization affects mostly neurons in TNC, while the whole-body allodynia is mediated by the central sensitization of third-order neurons, suggesting a thalamic involvement [87]. On a molecular level, the interictal levels of trigeminal CGRP are significantly elevated in patients with CM when compared to those with episodic migraine [88]. Interestingly, recent reviews proposed an interaction between CGRP and inflammation [89]. This process finally leads to increased production of pro-inflammatory mediators, which sensitize TG neurons [76, 90]. One example is the activation of TG neurons by peripheral (dural) inflammation [91], which mimics some of the features of CM.

NMDA-receptors, nitric oxide, and endogenous substances such as serotonin, bradykinin, substance P and CGRP are involved in the development of this central sensitization of the trigeminal nucleus and spinal dorsal nucleus [23, 24, 92]. Central sensitization, e.g. of the trigeminal nucleus will induce an increased pain transmission signal to the thalamus, limbic system and sensory cortex. The descending pathways of the rostral ventromedial medulla will facilitate the sensitized nociceptive second order neurons of the trigeminal nerve [71]. Although the majority of studies focused on migraine, the chronification mechanism of TTH seems to be not so different [71].

Sensitization has received the most interest in primary headaches and pathologies of the fifth cranial nerve. However, Groves and Thompson already in 1970’s proposed a “dual-process” theory [93]. The basis of this theory was based on the balance between depression (habituation) and facilitation (sensitization). Unlike sensitization, the neural mechanisms underlying habituation remain poorly understood [94].

Abnormal habituation patterns in migraineurs still lacks a definitive consensual interpretation. Nevertheless, there are some suggestions in the literature that central habituation could play a role in cluster headache (CH) and episodic migraine. For CH a habituation deficit of brainstem reflex responses has been observed [95]. Regarding episodic migraine, it was found that controls had a habituation response to repetitive sensory stimulation in contrast to migraine subjects. Therefore, it seems that amplified information processing from spinal trigeminal relay nuclei is linked to an impaired habituation response in migraineurs [96]. The cellular/physiological origin of these responses remains to be determined.

### Medication overuse headache

Medication overuse headache (MOH) is considered a secondary headache, with significant implications to primary headache sufferers. MOH is defined as a pre-existing headache (occurring at least 15 days/month) worsening due to regular overuse of medication (used > 10–15 days/month depending on the medication) for treatment of an acute or symptomatic headache for more than 3 months [97]. Medication overuse is the major risk factor for chronification in all primary headache forms, although the 80% of MOH patients have migraine as original primary headache, a smaller part TTH, and rarely post-traumatic headache [98].

Similarly to CM, in an MOH rat-model, persistent triptan exposure produced cutaneous allodynia and central upregulation of CGRP and neuronal nitric oxide synthase (nNOS) [99, 100]. Moreover, reduced serotonergic transmission seems to be involved in MOH development [101], possibly through a facilitation of the sensitization process via a maladaptive plasticity [98]. In humans, common neurophysiological investigation of central sensitization shows an abnormal cortical response to repetitive sensory stimuli, with an increased response amplitude after low numbers of stimuli [102] and a lacking habituation (which is instead normal in chronic migraineurs without MOH) [102], suggesting an altered plasticity.

A recent neurophysiological study investigating the serotonergic tone, found a low baseline serotonergic tone in chronic migraineurs with MOH, but it recovers after a week following anaesthetic block of the greater occipital nerve. Moreover, the size of the recover positively correlated with the clinical benefit after a month [103].

### TACs: cluster headache, SUNCT and SUNA

Trigeminal autonomic cephalalgias (TACs) are rare, but highly disabling primary headache disorders. The most common of the TACs is CH, known for its severely painful symptoms. Other subgroups of TACs are hemicrania continua (HC), paroxysmal hemicrania (PH), short-lasting unilateral neuralgiform headache with conjunctival injection and tearing (SUNCT) and short-lasting unilateral neuralgiform headache with cranial autonomic symptoms (SUNA).

The unifying pathophysiological mechanism for TACs is the role of the trigeminal autonomic reflex with parasympathetic activation and clinical presentation with strictly unilateral pain in the distribution of the trigeminal nerve and cranial autonomic features ipsilateral to the pain. Distribution of maximal pain in TACs is at the first branch of trigeminal nerve (V1) > upper cervical root (C2) > second branch (V2) > third branch (V3) [104].

Evidence for the peripheral mechanisms in CH include increased plasma levels of CGRP, PACAP [105] and vasoactive intestinal peptide (VIP) during acute cluster attack and even interictally [106]. Recently, a clinical study with a monoclonal antibody against CGRP was found positive in prevention of episodic CH [107]. Furthermore the SPG has connections to the trigeminovascular system, superior salivatory nucleus (SSN) and posterior hypothalamus: all areas that have an important role in the generation of CH attacks [108].

The last decade has brought more insight into pathogenesis of TACs, but still it is controversial whether the pain in TACs has a peripheral or central origin. Studies using animal models have shown that activation of trigeminal nerve may lead to activation of parasympathetic efferents, producing autonomic symptoms such as lacrimation, rhinorrhea and nasal congestion via the trigeminal-autonomic reflex. The origin of the cells for the parasympathetic autonomic vasodilator pathway is in the pontine SSN. The efferent projection is predominantly through the greater petrosal nerve, a branch of the facial nerve, and its projection through the SPG.

All primary headaches can be presented with autonomic symptoms to some degree, through reflex activation of the cranial autonomic outflow [109, 110]. A parasympathetic outflow activation probably results from stimulation of trigeminal afferents. In this trigeminal autonomic reflex, SPG may have a considerable role: in clinical studies, stimulation of SPG reduces intensity and frequency of CH pain [111]. The cyclical recurrence of the disorder (circadian and circannual rhythmicity), behavioural features, such agitation and restlessness, during acute cluster attacks, as well recently study on preictal and postictal symptoms in CH, led to theory of the key role of hypothalamus.

Genetics and neuroimaging studies has implicated that the brain and particularly the hypothalamus as a generator of TACs [109, 112]. Animal studies have shown that there are direct hypothalamic-trigeminal connections (trigeminohypothalamic tract), and bilateral descending hypothalamic projections to the spinal trigeminal nucleus [61]. Moreover, neuromodulation such as deep brain stimulation of posterior hypothalamus, occipital nerve stimulation, SPG stimulation has shown benefit to resistant chronic CH. Furthermore, cutting the trigeminal nerve root or ablative methods of TG does not resolve the pain in TACs [113].

In SUNCT and SUNA there are some similarities with trigeminal neuralgia (TN) that imply the involvement of neuropathic pain mechanisms, for example, the short-lasting unilateral attacks of pain, the cutaneous triggering and the response to antiepileptic medications [114]. TN will be covered below as we move on to conditions more plausibly linked to specific trigeminal nerve branches.

## Other painful conditionals of the trigeminal nerve branches

### The ophthalmic branch

Trigeminal Neuralgia (TN) is defined according to ICHD-3 criteria, as “recurrent unilateral brief electric shock-like pains, abrupt in onset and termination, limited to the distribution of one or more divisions of the trigeminal nerve and triggered by innocuous stimuli” [1]. The International Association for the Study of Pain (IASP) defines TN as “sudden, usually unilateral, severe, brief, stabbing, recurrent episodes of pain in the distribution of one or more branches of the trigeminal nerve” [115]. TN is a challenging syndrome and a common cause of head and facial pain, and usually along the distribution of the second or the third branch [116], therefore TN is covered in more detail below, as only a minority of cases of TN involves the first division of the trigeminal nerve.

Among the few secondary causes of headache in V1 are Tolosa-Hunt syndrome, orbital cellulitis, idiopathic intracranial hypertension and herpetic neuralgia. Damage to V1 can cause complex syndromes, as paratrigeminal oculosympathetic syndrome (Raeder’s syndrome) and recurrent painful ophthalmoplegic neuropathy (RPON) [117]. Raeder’s syndrome is a constant, unilateral pain caused by a disorder in the middle cranial fossa or of the carotid artery. RPON is an uncommon disorder with repeated attacks of paresis of one or more ocular cranial nerves (commonly the 3rd), with ipsilateral headaches [1]. The headache features are similar to typical migraine with frequent accompanying symptoms, such as nausea, vomiting photophobia and phonophobia. RPON is a diagnosis of exclusion. The differential diagnosis comprises all types of inflammatory or space-occupying lesions in the parasellar region and in the orbita [118].

One neuralgia that is linked to the V1 branch, is supraorbital neuralgia, characterized by persistent pain over the supraorbital region and medial region forehead [119]. It may be differentiated from supratrochlear neuralgia based on the topography of the pain, which can be confirmed with anaesthetic blockade [120]. Lacrimal neuralgia, is pain localized to the orbital and periorbital area, and was first described in 2013 [121]. All of these headaches are linked to V1, but little is known about their molecular pathophysiology. This is also the case for trochleodynia which is a spectrum of disorders characterized by pain arising from the trochlear region [122] and idiopathic ophthalmodynia [123] which is linked to pain in the eyeball.

Pain due to a cavernous sinus lesion, which is usually causing total ophthalmoplegia and being accompanied by a fixed, dilated pupil [124] or compression on the structures passing through the superior orbital fissure [125] can due to the anatomy also compromise the V1 branch. Furthermore, many cranio-cervical structures might present with facial pain and it is important always to be sure that the pain is not better accounted for by another diagnosis [1].

### The maxillary branch

Pain conditions linked to the V2 branch vary from mostly frequent TN to facial presentations of primary headaches. The diagnosis of TN is clinical and depends fundamentally on the description by the patient and characterization of pain [126]. TN is typically a unilateral condition, slightly, but significantly more frequent, on the right side [127]. Contrary to secondary forms, classical TN includes idiopathic cases as well as those caused by neurovascular compression, demonstrated by magnetic resonance imaging (MRI) or surgery, determining morphological changes to the trigeminal nerve root that represents about the 50% of cases. The exact extent of this neurovascular conflict needed to induce TN is still debated [128].

Classical TN may be purely paroxysmal, without concomitant continuous pain, or it may be with persistent background pain.

For TN, patients may describe a trigger point that elicits pain when touched: this could be interpreted as a manifestation of an erratic hyperactive functioning of the nerve. Furthermore, central causes have been proposed, even if it is difficult to determine which of the these changes are cause and effect: volume reduction in somatosensory cortex, thalamus and other subcortical areas has been observed [129], as well as functional connectivity alterations were described in sensory trigeminal pathways [130]. Sometimes trigeminal nerve atrophy can be demonstrated in patients with TN by high-resolution imaging and it is significantly correlated with the severity of neurovascular compression [131].

Another important cause of facial pain in the V2 territory, which has been considered “the atypical counterpart to trigeminal neuralgia” [132], is the persistent idiopathic facial pain (PIFP), previously termed atypical facial pain or atypical odontalgia when occurring in the oral cavity. PIFP is defined as a continuous facial pain, typically localized in a circumscribed area of the face, which is generally not accompanied by any neurological or other lesion identified by clinical examination or clinical investigations [132]. This facial pain, which occurs daily and persists throughout the day, is generally described as deep, poorly localized, and is not associated with sensory loss or other neurological deficits, which differentiates it from a pure neuropathic process. The pathophysiology is not fully elucidated and possibly it relies on a combination of neuropathic pain, central sensitization, and local inflammation [132–134].

This complex pathophysiology is reflected by the difficulty in treating PIFP successfully, and the concept that different types of interventions are needed [135]. While the large majority of case are idiopathic with investigations including X-ray of the face and jaws or cranial computed tomography (CT) or MRI not demonstrating any relevant abnormality, a part of PIFP-like disorders can be secondary to dental or oral conditions [136–138].

Lastly, neuralgia of the infraorbital nerve (numb cheek syndrome) is an unusual cause of facial pain, most often associated with the V2 branch [139]. The pain can be characterized by constant discomfort, often in the form of stabbing pain, often accompanied with hypersensitivity to palpation in the infraorbital notch [140], and can be linked to an underlying cancer [141].

### The mandibular branch

A trigeminal nerve injury that mainly affects its V3 branch is characterized by acute paroxysmal painful episodes [142, 143] with a sudden onset that may involve all the aforementioned structures [144]. The typical associated symptoms are PIFP or burning mouth like syndrome (BMLS) [145]. While PIFP can affects either V2 or V3, with a preference for the former, BMLS is defined as a multifactorial chronic pain condition characterized by a burning or stinging sensation, often accompanied by xerostomia and preferably located on the tongue or, in a lesser extent, other specific areas of the mouth, in a clinically healthy oral mucosa [146]. The epidemiology varies from 0.01% to 40% according the studies, generally observed in middle-aged patients and postmenopausal women [147, 148].

No definitive aetiology has been established for burning mouth syndrome, an intramural burning sensation for which no medical or dental cause can be found: both central and peripheral nervous systems seem to be involved and some studies suggested a trigeminal small fibre sensory neuropathy in innervation territory of maxillary nerve [149]. The diagnosis is generally reached after a series of tests, including neurophysiological evaluation and peripheral lingual nerve anaesthetic block, allowing the distinction between peripheral and central forms (for a review, see [150]). Therapeutic options remain however low, with only topical or systemic low-dose clonazepam as a valuable treatment [151, 152]. Topical capsaicin or saliva substitute are second line options in peripheral forms, while amitriptyline or gabapentin are considered in central form [150].

One of the most “dangerous” neuralgias is the “Numb chin syndrome” which can occur from a lesion anywhere along the course of the trigeminal nerve. Typically it represents loss of the terminal and sensory branch of the mandibular branch and is often linked to cancer, such as metastatic tumours [153].

Finally, temporomandibular disorders are also linked to the V3 branch [154]. TN can be differentiated from temporomandibular joint dysfunction by the acute, piercing, and stabbing nature of neuralgic pain occurring at a single facial location, spreading along the course of the nerve on one side, leading to differences in the character and intensity of the pain [155].

## Treatments targeting the trigeminal nerve

### Acute treatments

Although many new therapeutic targets are under investigation [156], the most frequently used acute treatment for headaches are non-specific drugs, such as NSAIDs.

**Cyclooxygenase 1 and 2 (COX-1, COX-2) inhibitors** have peripheral effect on prostaglandin synthesis involved in inflammatory processes. Acetylsalicylic acid is found to have additional inhibitory effect on the central trigeminal neurons after sagittal sinus stimulation [157]. Ketorolac (a nonselective COX-inhibitor) was found to prevent sensitization at the trigeminal nucleus neurons [158]. COX-2 inhibitor piroxicam showed good effect in PH and celecoxib in HC [159].

It has been shown that some TACs (e.g. PH) respond well to indomethacin. In a case study, the patient became pain-free overnight after the use of indomethacin after 12 years of failed treatments [160]. Indomethacin is a COX-inhibitor that inhibits evoked firing in the TCC in animal models [110]. Furthermore, indomethacin exerts an effect on IL-1β induced prostaglandin E synthesis via COX-2 in cultured rat trigeminal cells. Consequently to the blockage of prostaglandin E release, the release of CGRP was inhibited [161, 162]. According to studies, indomethacin has higher odds of responders and complete responders than any other treatment option in HC and PH [159]. On the other hand, indomethacin can also inhibit NO-induced vasodilatation. Contrary to NO-induced CH-like and migraine-like headache that start after a certain delay from NO administration, NO-induced PH symptoms begin immediately after the administration, and this can be the reason behind the different effectiveness of indomethacin [163].

Furthermore, there are a subcategory of headaches that seem to response well to indomethacin, so called “Indomethacin-responsive headaches”. These are sexual headache, trocheodynia, Valsalva-induced headache, primary stabbing headache, hypnic headache and primary exertion headache (also called exercise headache) [164]. This could be linked to inhibitory effect of indomethacin on trigeminal nociceptive firing and the trigeminoautonomic activation, which has been shown in animals by Akerman et al. [165].

**Ergotamine and dihydroergotamine** were the first specific acute antimigraine drugs in use for several decades [166]. Ergot alkaloids are non-specific 5-HT_1_ receptor agonists that also bind α-adrenoceptors and dopamine receptors. Therapeutic effect of these drugs likely originates from their agonist properties at 5-HT_1B_ and 5-HT_1D_ receptors that lead to trigeminal inhibition by, for example, reducing CGRP release [167, 168]. Other previously proposed antimigraine mechanisms include constriction of large capacitance arteries, closure of arteriovenous anastomoses, inhibition of neurogenic inflammation, and blockade of transmission in the TNC [169].

**Triptans** have been studied in the context of headaches for decades. They are potent 5-HT_1B/1D_ receptor agonists, a majority of them are also 5HT_1F_ receptor agonists [170]. There is evidence that triptans exert their clinical effect peripherally by binding to 5-HT_1B_ receptors, resulting in slight vasoconstrictive properties as well as blocking CGRP release and centrally by blocking trigeminal transmission through binding at 5-HT_1D_ receptors in the trigeminal nuclei of the brainstem [171]. In addition to being important in treating migraine, triptans (especially subcutaneous sumatriptan) are considered the most effective treatment in cluster headaches [172]. Subcutaneous sumatriptan was reported to decrease pain significantly also in TN [173]. In clinical practice, triptans are preferred to ergotamine derivatives, because they are at least as potent, with better tolerability and fewer side effects [158].

**Ditans** are selective 5-HT_1F_ receptor agonists that were developed in hope to increase the effectiveness and to lower the risk of cardiovascular side effects of triptans. 5-HT_1F_ receptors are located in both peripheral and central sensory trigeminal neurons, and their activation is found to hyperpolarize nerve terminals inhibiting trigeminal impulses [174], inhibit the CGRP-mediated vasodilation in vivo, modulate the pain perception pathway and prevents CGRP release [170, 175]. Lasmiditan is the only compound of this drug class that has been evaluated in Phase III clinical trials and approved by the FDA [176]. It penetrates the BBB and could thus exert effects centrally, in addition to the trigeminovascular system [174].

**Gepants** antagonize the CGRP receptor in trigeminal system. These drugs were promising in trials but were discontinued due to low oral bioavailability (olcegepant) and unexpected hepatotoxicity (telcagepant) [177]. However, ubrogepant and rimegepant tablets have both recently received FDA approval (23rd December 2019 and 27th February 2020) for acute treatment of migraine in adults [178].

**Sodium channel blockers**, such as lidocaine, blocks sodium channels in a frequency-dependent and voltage-dependent manner. The nerve block with lidocaine stops the nociceptive firing and the neuronal hyperexcitability in first order neurons reducing peripheral sensitivity [179]. Intranasal lidocaine administered ipsilaterally to the pain to anaesthetize the SPG, which is responsible for the autonomic symptoms associated to TACs or other headaches via the trigemino-autonomic reflex [180]. Intranasal lidocaine is considered a second line treatment for CH [181]. The most effective treatment of SUNCT/SUNA acute attacks is considered to be intravenous lidocaine, subcutaneous treatment also can be used [159]. Furthermore, TN studies have shown that lidocaine, rubbed onto the trigger zone of the oral mucosa, provided a few hours of pain relief [173].

Some voltage-sensitive sodium channel blockers, such as lamotrigine or amides (carbamazepine, oxcarbazepine, eslicarbazepine), are often the first line treatment for some painful conditions affecting the trigeminal nerve, and most likely they act by stabilizing neural membranes and inhibit the release of neurotransmitters [182]. In a Cochrane review (from 2013) it was concluded that there was no reduced headache frequency from carisbamate, clonazepam, lamotrigine, oxcarbazepine, pregabalin, or vigabatrin [183]**.** However, carbamazepine has shown some effect in familial hemiplegic migraine [184]. Further studies are needed to determine the efficacy of the newer drugs [185].

**High-flow oxygen** is considered a first line treatment for CH [172, 181]. The mechanism of action of oxygen in now thought to be related not to its vasoconstrictor effect, but rather to the inhibition of neuronal activation in the TNC [186]. Oxygen is also thought to normalize the CGRP levels and thus reduce the activity in the trigeminovascular system [106].

### Prophylactic treatments

CGRP released from trigeminal terminals results in vasodilation via CGRP receptors on the smooth muscle cells of meningeal and cerebral blood vessels [187] and activation of Aδ-fibres, with the possibility of inducing sensitization [188]. Although antibodies can theoretically target CGRP or its receptors in the brain regions, the BBB permeability is low [189, 190]. Therefore their therapeutic action may be entirely peripheral and likely affecting targets within the trigeminovascular system [191, 192].

**Monoclonal antibodies** acting on CGRP pathway, with indications for migraine prevention, have been developed in recent years: one targeting the CGRP receptor (erenumab) and three targeting the CGRP peptide (eptinezumab, fremanezumab and galcanezumab) [193]. Fremanezumab inhibits activation of central trigeminovascular neurons with input from the intracranial dura, but not the facial skin or cornea [194] providing evidence that antibodies against CGRP can inhibit trigeminal neuron activation. However, their site of action along the trigeminal pathway remains uncertain, though recently, axon-axon signalling at the node of Ranvier between C- and Aδ-fibres was suggested as a plausible site of action [167]. A role for the trigeminal nerve in CH and PH is indicated by the increased concentrations of CGRP in the ipsilateral jugular vein during attacks [106, 195]. Galcanezumab was recently reported to reduce the frequency of episodic CH attacks [107].

**Onabotulinumtoxin A**, beta blockers (e.g propranolol), tricyclic antidepressants (e.g amitriptyline), anticonvulsants (e.g topiramate) and calcium channel blockers (e.g flunarizine) continue to be standard therapies for migraine prevention [196–198]. Though mainly known for its therapeutic effects in CM, onabotulinumtoxin A has been shown positive results in treating TN [199] and refractory, chronic CH [200]. Onabotulinumtoxin A modulates neurotransmitter release, changes in surface expression of receptors and cytokines as well as enhancement of opioidergic transmission [201]. This is done by cleaving synaptosomal nerve-associated protein 25 (SNAP-25), a vesicle docking protein, within the cell and thus disrupting the fusion of neurotransmitter vesicles to the synaptic cleft [202]. It is likely that onabotulinumtoxin A reduces both peripheral and central sensitization through such mechanisms [203, 204].

### Non-pharmacological treatments

There are currently several non-invasive and invasive stimulation techniques that may help patients who wish to avoid, are refractory to or intolerant of previous drug therapies [108]. Non-invasive stimulation options include the supraorbital stimulation, vagus nerve stimulation (VNS) and the single-pulse transcranial magnetic stimulation [108].

The initial use of VNS to treat headaches first came from the epilepsy field, following several anecdotal reports of migraine improvement in patients with comorbid epilepsy who had been implanted with the device [205]. The vagal nerve is a mixed motor and sensory nerve that is important in controlling autonomic responses; it projects to several higher centres that are important in pain regulation [108]. Indeed, VNS was sufficient to significantly inhibit nocifensive head withdrawal response from mechanical stimulation of V1 trigeminal nociceptors [206]. The commercial use in migraine therapy certainly came with the development of portable devices, which allow to stimulate the vagal nerve transcutaneously at the neck (GammaCore® device) or in its auricular portion (Nemos® device) in a non-invasive way [205]. Possible uses for VNS is preventative treatment of CH, acute treatment of CH [207] and preventive treatment of CM (controlled studies are needed to investigate this point) [208].

The occipital nerves are a target for stimulation due to the anatomical overlap between the trigeminal and cervical afferents in the TCC [108]. This allows stimulation of the occipital region to modulate pain in the trigeminal distribution. Occipital nerve stimulation (ONS) is a surgical procedure where electrodes are placed subcutaneously in the occipital region and then wired to a battery pack in the chest or abdomen [108]. Open-label studies have shown possible efficacy in preventing CM, chronic CH [209]. Possible uses for ONS is preventative treatment of refractory CM and chronic CH [108].

**Trigeminal radiofrequency thermocoagulation** (TRT) is a surgical intervention used for the treatment of TN. TRT involves puncturing the TG or its branches with a CT- or X-ray-guided a radiofrequency thermocoagulation (RFT) ablation needle [210]. Sensory and motor stimulation are used to replicate the patient’s pain and locate and destroy the responsible nerve. Recurrence is possible after RFT ablation; some patients need to continue medication treatment, while others may require reoperation, and postoperative facial numbness is a notable problem combined with developing neuropathic pain [210].

**Peripheral nerve blocks** (PNB) have been used for the acute and preventive treatment of a variety of primary headache disorders [211]. PNB are generally safe and well-tolerated procedures that may be performed in the outpatient setting [211]. PNB can be used in primary (migraine, CH, and nummular headache) and secondary headaches (cervicogenic headache and headache attributed to craniotomy), as well in cranial neuralgias (trigeminal neuropathies, glossopharyngeal and occipital neuralgias) [179]. This procedure can be necessary for both diagnosis and treatment (e.g a PNB of the inferior dental plexus will halt the pain caused by TN but not a temporomandibular disorder), while in cases it is considered an adjuvant treatment [179]. Interestingly, a retrospective case-study reported long-lasting (1–8 months) and immediate pain-relief for refractory TN patients treated with PNB [212]. This surprisingly long-lasting result (as the half-life of anaesthetics is usually brief) could be due to the dose-dependent neurotoxicity of local anaesthetics [213].

The block of the greater occipital nerve with an anaesthetic and corticosteroid compound has proved to be effective in the treatment of CH. Regarding the treatment of other headaches and cranial neuralgias, controlled studies are still necessary to clarify the real role of peripheral nerve block [179].

Although nummular headache is characterized by continuous pain in a small circumscribed area, it surprisingly does not respond well to PNB [214], this contrasts to the onabotulinumtoxin A, which seems effective [215]. This suggests that there is difference in the mechanism of a nerve block, and the use of onabotulinumtoxin A.

## Conclusion

The involvement of the fifth cranial nerve in headache has been thoroughly established, following the original postulation by Wolff in the 1940’s. The current review summarizes the anatomical and physiological link between headaches, pain perception and the fifth cranial nerve. The most striking evidence comes from the numerous treatments available, where their targets are almost exclusively found in the nerves of trigeminal ganglion; the hub of the fifth cranial nerve. Although we believe that the headache-trigger most likely have the origin in the CNS, this review underscores the importance of trigeminal neurons in the perception of pain. Only when the activation of the fifth cranial nerve is combined with knowledge of central pathological mechanisms, we can start to fully understand the pathology of headache.

## Data Availability

Not applicable.

## Abbreviations

CGRP: Calcitonin gene-related peptide
BBB: Blood-brain barrier
TG: Trigeminal ganglion
TN: Trigeminal Neuralgia
WHO: World health organization
TTH: Tension-type headache
MOH: Medication-overuse headache
CN V: Fifth cranial nerve
V1: Ophthalmic branch
V2: Maxillary branch
V3: Mandibular branch
NGU: Neuronal and glia unit
ECM: Extracellular matrix
SGC: Satellite glial cell
PACAP: Pituitary adenylate cyclase-activating polypeptide
TCC: Trigeminocervical complex
TNC: Trigeminal nucleus caudalis
PC: Pars Caudalis
PO: Pars Oralis
PI: Pars Interpolaris
TACs: Trigeminal autonomic cephalalgias
RPON: Recurrent painful ophthalmoplegic neuropathy
MRI: Magnetic resonance imaging
PIFP: persistent idiopathic facial pain
CT: computed tomography
BMLS: burning mouth like syndrome
TRP: Transient receptor potential
TRPV1: TRP vanilloid 1
TRPA1: TRP ankyrin 1
TRPM8: TRP melastatin 8
GWAS: Genome wide association studies
ICHD: International Classification of Headache Disorders
NMDA: N-methyl-D-aspartate
nNOS: neuronal nitric oxide synthase
CH: Cluster Headache
HC: Hemicrania continua
PH: Paroxysmal hemicranias
SUNCT: Short-lasting unilateral neuralgiform headache with conjunctival injection and tearing
SUNA: Short-lasting unilateral neuralgiform headache with cranial autonomic symptoms
C2: Upper cervical root
SSN: Pontine superior salivatory nucleus
COX: Cyclooxygenase
IL-β: Interleukin 1 beta
NO: Nitric oxide
5-HT: 5-hydroxytryptamine
FDA: U.S Food and Drug Administration
SNAP-25: Synaptosomal nerve-associated protein 25
VNS: Vagus nerve stimulation
ONS: Occipital nerve stimulation
TRT: Trigeminal radiofrequency thermocoagulation
RFT: Radiofrequency thermocoagulation
PNB: Peripheral nerve blocks
